# Antisense miR-132 blockade via the AChE-R splice variant mitigates cortical inflammation

**DOI:** 10.1038/srep42755

**Published:** 2017-02-17

**Authors:** Nibha Mishra, Lyndon Friedson, Geula Hanin, Uriya Bekenstein, Meshi Volovich, Estelle R. Bennett, David S. Greenberg, Hermona Soreq

**Affiliations:** 1 The Edmond and Lily Safra Center for Brain Sciences, The Hebrew University of Jerusalem, The Edmond Safra Campus, Givat Ram, Jerusalem 9190401, Israel; 2 The Silberman Institute of Life Sciences, The Hebrew University of Jerusalem, The Edmond Safra Campus, Givat Ram, Jerusalem 9190401, Israel

## Abstract

MicroRNA (miR)-132 brain-to-body messages suppress inflammation by targeting acetylcholinesterase (AChE), but the target specificity of 3’-AChE splice variants and the signaling pathways involved remain unknown. Using surface plasmon resonance (SPR), we identified preferential miR-132 targeting of soluble AChE-R over synaptic-bound AChE-S, potentiating miR-132-mediated brain and body cholinergic suppression of pro-inflammatory cytokines. Inversely, bacterial lipopolysaccharide (LPS) reduced multiple miR-132 targets, suppressed AChE-S more than AChE-R and elevated inflammatory hallmarks. Furthermore, blockade of peripheral miR-132 by chemically protected AM132 antisense oligonucleotide elevated muscle AChE-R 10-fold over AChE-S, and cortical miRNA-sequencing demonstrated inverse brain changes by AM132 and LPS in immune-related miRs and neurotransmission and cholinergic signaling pathways. In neuromuscular junctions, AM132 co-elevated the nicotinic acetylcholine receptor and AChE, re-balancing neurotransmission and reaching mild muscle incoordination. Our findings demonstrate preferential miR-132-induced modulation of AChE-R which ignites bidirectional brain and body anti-inflammatory regulation, underscoring splice-variant miR-132 specificity as a new complexity level in inflammatory surveillance.

Most coding RNA transcripts undergo alternative splicing^1^, and the resultant splice variants can interact with non-coding microRNAs (miRs) which suppress their function^2^. Functional miR recognition sites mainly reside within and near both ends of the 3′-untranslated region (3′-UTR)^3^. In over half of the human genes, alternative splicing events modify the 3′-UTR^4^,^5^, indicating functional association of transcript variants with miRs that target them^6^. Such 3′-UTR alternative splicing may further produce protein isoforms with modified properties, as is the case for the secreted, soluble monomeric acetylcholine hydrolyzing protein AChE-R, which differs in location from the synaptic membrane-associated AChE-S tetramers^7^. However, whether miRs differentially suppress these splice variant products of the AChE gene, and if so what are the consequences of such selectivity remain unknown. For example, miRs modulate neurotransmission and immune pathways in body and brain in response to acute inflammation and stressful experiences^8^,^9^,^10^. This induces a rapid anti-inflammatory response which is initiated by potentiated cholinergic tone via brain-to-body signaling of the vagus nerve^11^. We, and others have shown that miRs as well as natural antisense transcripts^12^ are pivotal for controlling these neuronal-immune processes^13^,^14^,^15^ and provide continuous surveillance over peripheral cholinergic anti-inflammatory signaling^16^,^17^,^18^,^19^. Nevertheless, whether the body orchestrates this brain-induced miR-mediated response via splice variant preference was not yet explored.

Notably, miRs operate in a complex combinatorial mode, driven by simultaneous cell type and tissue-specific, context-dependent suppression of multiple target transcripts^18^,^20^. Also, numerous transcripts are commonly suppressed by multiple miRs^18^,^19^, and recent work supports the concept that miRs migrate between tissues when packaged in exosomal lipoprotein compartments^21^. Yet, some miRs show more potent function than others; in particular, miR-132 plays multiple roles in neuronal development, anxiety, and metabolism. It suppresses many pro-inflammatory target transcripts, including the transcriptional co-activator P300^22^, the growth factor HB-EGF^23^, the dopaminergic regulator Nurr1^24^ and the epigenetic modulator SIRT1, further potentiating anti-inflammatory processes^25^. We set out to explore the hypothesis that miR-132 is causally involved in the bidirectional brain-body signaling that provides continuous surveillance over inflammatory reactions. Since anti-inflammatory responses involve elevation of the brain’s miR-132^26^,^27^, and as luciferase tests and lentivirus suppression validated that miR-132 targets AChE^26^,^27^, we predicted that miR-132 conveys brain-to-body anti-inflammatory signals, by modulating acetylcholine (ACh) levels via targeting, and suppressing both central and peripheral AChE^26^. This inhibition limits ACh hydrolysis, and elevates the cholinergic tone (known to increase substantially under acute stress)^28^. Moreover, miR-132 may induce, rather than suppress particular pathways; for example, by decreasing the expression of negative transcriptional regulators known to exist in the cholinergic system^29^. Therefore, increasing levels of brain miR-132 can attenuate inflammation through up- and down-regulation of multiple genes and pathways.

Cholinergic perturbation-induced alternative splicing promotes exon inclusion, which shifts production of the major ‘synaptic’ membrane-associated AChE-S tetramers to the soluble AChE-R monomer^28^,^30^,^31^. The 3′-untranslated regions of both AChE variants include the ‘seed’ motif for miR-132^32^, indicating that stress-induced miR-132 increases can suppress both AChE-S and AChE-R. However, AChE-S suppression would elevate synaptic ACh levels and modulate cholinergic neurotransmission, whereas AChE-R suppression would elevate extracellular ACh, potentiating the cholinergic anti-inflammatory effect^11^. In comparison, acute organophosphate poisoning blocks AChE irreversibly, and induces cholinergic hyper-excitation while leading to massive inflammation^33^. To determine if the AChE-S or AChE-R variants are preferentially targeted by miR-132, we used surface plasmon resonance (SPR) to predict variant binding preference; and employed an antisense oligonucleotide suppressor of miR-132, AM132, which cannot cross the blood brain barrier and whose effect is limited to the periphery^34^, compatible with numerous reports^35^. To find if such suppression involves brain-body inflammatory regulating messages^36^, we characterized the brain and body reaction of mice injected with AM132 or LPS (known to increase miR-132 expression^26^) by RNA-sequencing, qRT-PCR validations, AChE activity tests and bioinformatics interrogation (see Fig. 1 for scheme of workflow).

## Results

### SPR-based analysis predicts tighter miR-132 interaction with the soluble AChE-R splice variant

Like many other miRs, miR-132 has an exhaustive set of potential targets (see miRNAwalk: http://www.umm.uni-heidelberg.de/apps/zmf/miRNAwalk). Of those, we were particularly interested in the validated miR-132 targets AChE-S and AChE-R^32^ as well as SIRT1^37^, all of which may be involved in anxiety and anti-inflammatory signaling. To experimentally measure association of miR-132 with its targets, we used a SPR binding assay. Given that miRNA-target interactions may involve longer regions than the seed itself^38^, we immobilized biotinylated 30-mer RNA sequences corresponding to the 3′-UTR regions of the major AChE-S splice variant^7^ or of the soluble AChE-R splice variant to SPR chips and injected a 22-mer RNA oligonucleotide containing the miR-132 sequence (see Supplementary Fig. S1 and Supplementary Table S1). Results demonstrated a ~2.5-fold higher affinity of miR-132 to the stress-inducible minor splice variant AChE-R compared to the major synaptic AChE-S sequence (*K*_D_ of 8.13 vs 18.75 nM, Fig. 2a,b) and an even higher affinity to SIRT1 (0.42 nM, Fig. 2b), predicting a hierarchical binding preference of miR-132 to (in increasing affinities) SIRT1, AChE-R, and AChE-S. Interestingly, all three showed similar affinity to that of the other AChE-targeting miR-608^39^.

### AM132 suppresses peripheral miR-132 and preferentially elevates AChE-R activities

**Figure 1:**
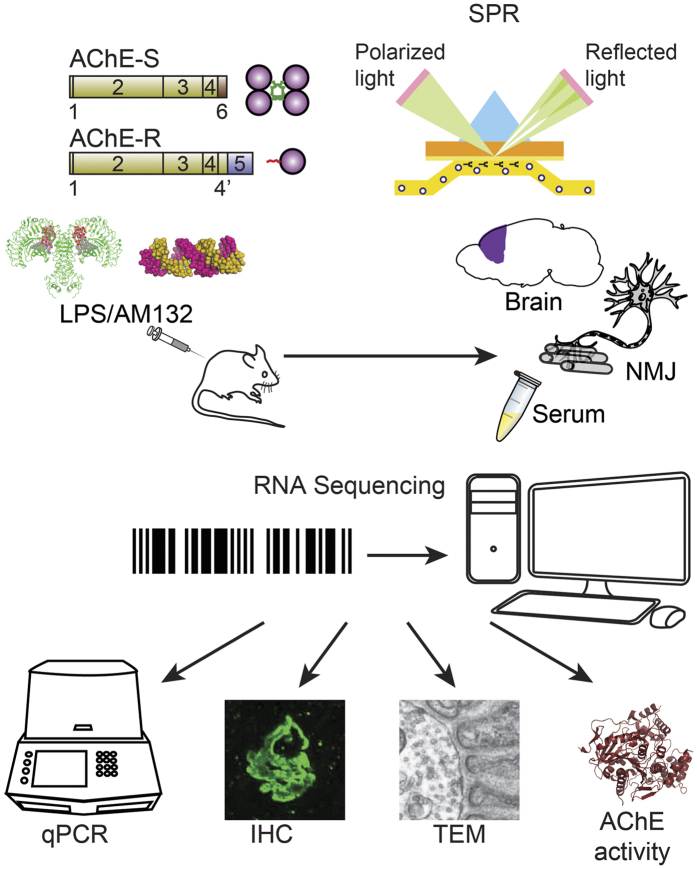
Schematic work flow. We injected LPS (i.p.) and AM132 (i.v.) on two alternate days and 24 h later extracted brain, muscle, and serum. We used SPR tests to assess variant specificities; RNA-sequencing to derive frontal cortices miR profiles; and bioinformatics processing and qPCR for quantifying mRNA targets of miR-132 and pro-inflammatory cytokines. AChE activity was measured in serum, tissue homogenates and NMJs, and NMJ AChE and nAChR were labeled with histochemical Karnovsky’s staining; immunohistochemistry and NMJ anatomy were explored by light and transmission electron microscopy (TEM), respectively.

**Figure 2:**
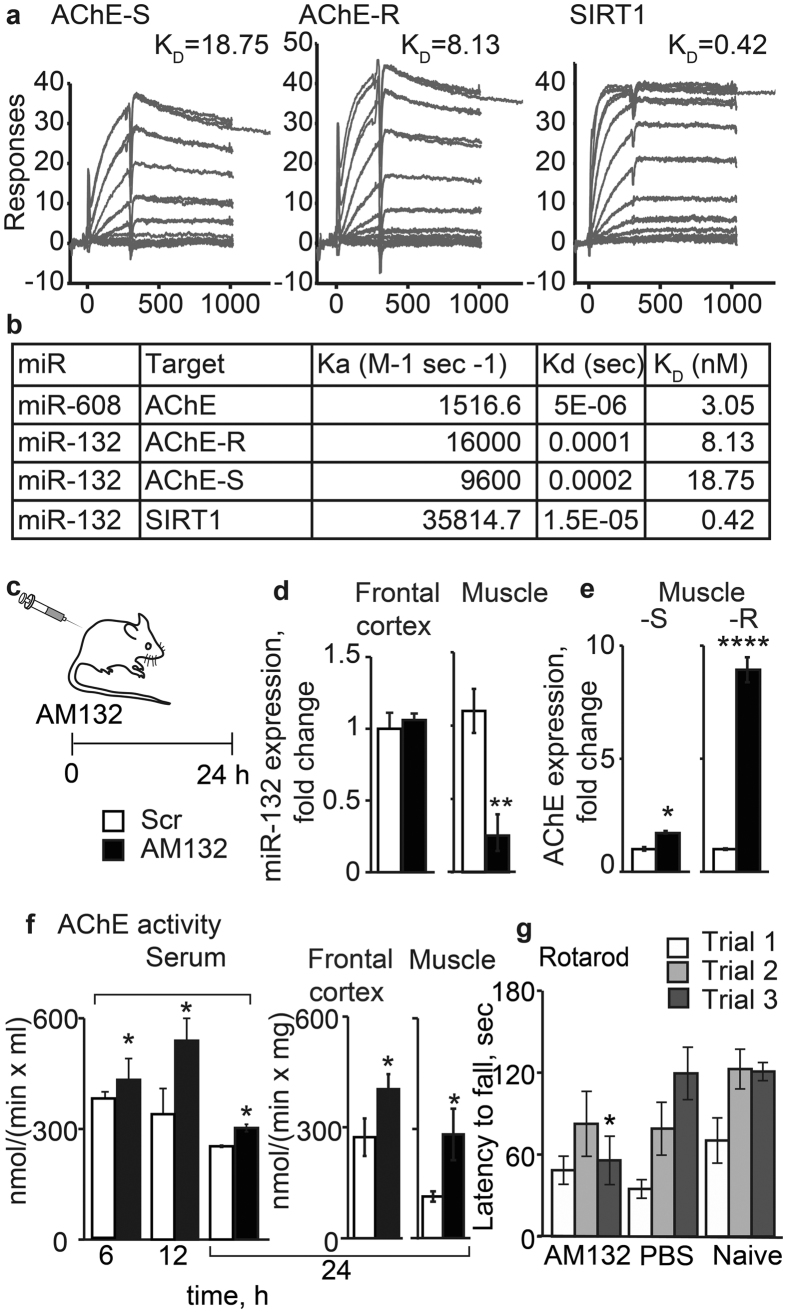
AM132 reduces miR-132 and elevates AChE in periphery and brain. (**a,b**) SPR quantifications predict Ka (M-1 sec-1), Kd (sec) and KD (nM) binding affinities of miR-132 to AChE-S, AChE-R and SIRT1. (**c**) Schematic work flow of AM132 treatment. (**d**) MiR-132 levels are unchanged in the brain and significantly decreased in muscle (p < 0.01, t-test). (**e**) Elevated levels of the AChE-R (p < 0.0001) and AChE-S (p < 0.01) alternative splice variants in AM132-treated gastrocnemius muscle as compared to control. (**f**) Increased serum AChE activity post-AM132 treatment at 6, 12 and 24 h (p < 0.05, T-test); and at 24 h in frontal cortex and in muscle (p < 0.05). (**g**) AM132 treated mice show early latency to fall off the bars in a third trial of a fixed speed Rotarod test (*p < 0.05, ANOVA).

Inflammation commonly induces ‘sickness behavior’^36^,^40^, suggesting that transcriptional changes in the Central Nervous System (CNS) of miR and mRNA expression during inflammation may be causally involved. To address this issue, we treated mice with AM132, a 15-mer LNA-protected antisense oligonucleotide (Figs 1, 2c) that selectively binds and inactivates miR-132, or with a control oligonucleotide with no murine transcript targets (Fig. 1, Supplementary Fig. S1). Muscle, but not cortical miR-132 levels were reduced (Fig. 2d) within 24 hours in AM132-treated mice, compatible with the predicted failure of AM132 to cross the blood-brain barrier^34^. Also, q-PCR measurements revealed elevated muscle AChE-R, much more than AChE-S mRNA levels in AM132-treated mice (n = 4 Fig. 2e; p < 0.0001 compared to p < 0.05), and the effect was even greater than what was predicted by the SPR measurements, suggesting involvement of additional elements in the *in vivo* reaction. Given that alternative splicing regulators may control this change in AChE splice variants, we further quantified the muscle mRNA encoding the SC35 splicing factor known to shift the 3′-UTR of AChE-S to AChE-R^41^. Muscle SC35 mRNA levels increased by approximately two fold (Supplementary Fig. S2a), which might only explain part of the increase in AChE-R^41^. In the frontal cortex, SC35 levels remained unchanged under AM132, but were significantly elevated under LPS exposure. In the frontal cortex, SC35 levels remained unchanged under AM132, but were significantly elevated under LPS exposure (Supplementary Fig. S2b). Additionally, SIRT1 expression was modestly increased (Supplementary Fig. S2c), further highlights the pronounced AM132 impact on AChE-R. Compatible with the elevated AChE-R mRNA levels, the ACh hydrolytic activity of AChE was increased in the serum, brain and muscle of AM132-treated mice at 6, 12 and 24 hours post-injection (Fig. 2f; n = 4, p < 0.05), in spite of the brain-inaccessible nature of AM132^35^. However, in spite of this massive elevation in ACh hydrolysis, AM132-treated mice only showed mild muscle incoordination, evident in their shorter latency to falling off a beam (n = 6, p < 0.05, ANOVA) on the third trial in the Rotarod test. This observation reflects somewhat exacerbated muscle fatigue compared to PBS-treated and naïve mice (Fig. 2g). Together, these findings validated a preferential impact of peripherally modified miR-132 signaling via the soluble AChE-R splice variant and consequently weakened cholinergic signaling, indicating that selective AM132 modulation of muscle AChE-R expression leads to mild muscle incoordination.

### AM132 and LPS exert inverse impacts on cortical cholinergic anti-inflammatory regulators

**Figure 3:**
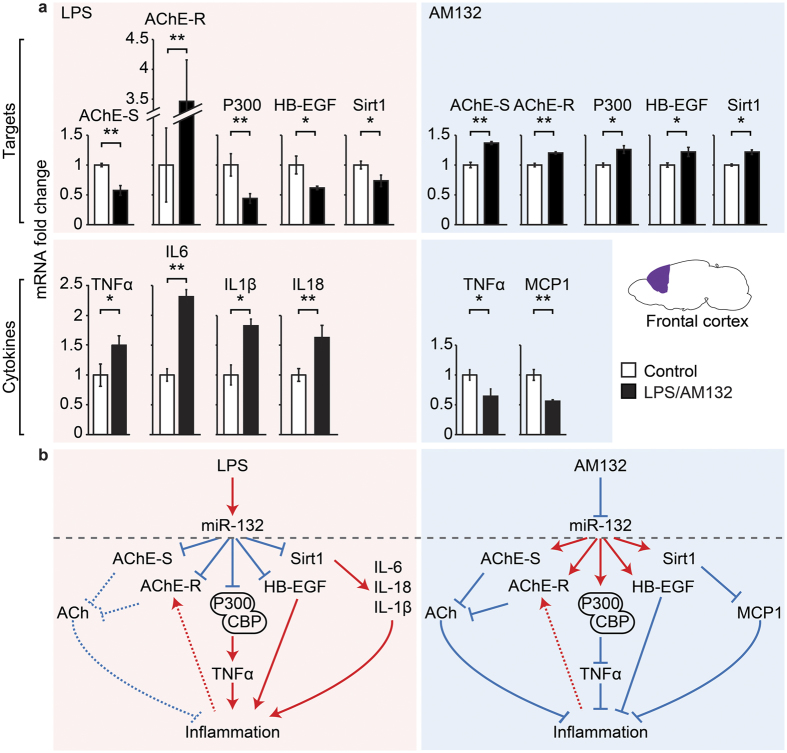
Inversely modulated cortical targets and cytokines under LPS and AM132 treatment. (**a**) qRT-PCR of targets (top) and pro-inflammatory cytokines (bottom) under LPS (left) and AM132 treatment (right). Note inverse regulation under LPS exposure (pink background) and AM132 treatment (blue background). P values reflect 0.05 (*) or 0.01 (**) (Student’s T test). Note that LPS exposure elevates (red arrows) miR-132, reducing its targets (blue lines) and enhancing the consequent inflammation biomarkers. (**b**) Schematic representation of the tested brain and muscle tissues, the alternative splicing of AChE mRNA and the observed changes in modified miR-132 targets and pro-inflammatory cytokines. AChE-S downregulation may mitigate cholinergic neurotransmission whereas AChE-R down regulation should enhance the cholinergic anti-inflammatory reflex, demonstrating bidirectional rebalancing. In comparison, AM132 treatment (blue background scheme) upregulates miR-132 targets which downregulates pro-inflammatory cytokines, with elevated AChE-S and AChR-R inhibiting the cholinergic anti-inflammatory effect.

Peripheral elevations and decreases in miR-132 targets may modify efferent vagus signaling reaching the brain^11^,^26^, which would affect the brain’s cholinergic balance and inflammatory state. To quantify miR-132 target transcripts, we compared frontal cortices of AM132-treated and LPS-exposed mice (shown to elevate brain miR-132 levels, Supplementary Figs S3, S4). Cortices from LPS-exposed mice presented down-regulation of the ‘synaptic’ variant AChE-S (n = 8, p < 0.01), the transcription controller P300 (n = 8, p < 0.01), the epigenetic modulator SIRT1 (n = 8, p < 0.05), and the growth factor HB-EGF (n = 8, p < 0.05), which are all validated miR-132 targets (Fig. 3, left hand side), compatible with our previous findings that LPS exposure modulated cortical cholinergic neurotransmission via potentiating miR-132 suppression^26^,^42^. In contrast, the stress-induced soluble AChE-R variant and the cholinergic-regulated pro-inflammatory cytokines IL18 (n = 8, p < 0.01), IL6 (n = 8, p < 0.01), IL1β (n = 8, p < 0.05), and TNFα (n = 8, p < 0.05)^11^,^26^ were all elevated, reflecting an accentuated cortical inflammatory state following LPS exposure. These findings, and that the splice factor SC35, which promotes the alternative splicing shift to AChE-R was also increased under LPS exposure (Supplementary Fig. S2b), together indicated co-exacerbated brain anxiety and inflammation under LPS exposure.

Unlike the LPS effect, cortices from AM132-treated mice showed significant up-regulation of both the membrane-bound AChE-S (n = 4, p < 0.01) and the soluble extracellular AChE-R transcripts, predicting suppressed cholinergic signals and modified structure and function of cholinergic synapses^43^. In parallel, up regulation of P300 (n = 4, p < 0.05), SIRT1 (n = 4, p < 0.05), and HB-EGF (n = 4, p < 0.05) indicated a synergized brain anti-inflammatory impact of AM132, along with potentiated individual functions of these target genes^13^,^16^,^17^. This complex outcome was accompanied by downregulation of the pro-inflammatory MCP-1 (n = 4, p < 0.01) and TNFα (n = 4, p < 0.05) cytokines (Fig. 3, top right). Thus, in spite of its brain-impermeable feature, peripheral miR-132 manipulation affected both the miR-132 targets and their downstream cortical transcripts, in a largely inverse manner to the LPS effect (Fig. 3, bottom schemes).

### AM132 and LPS induce inversely oriented changes in cortical miR profiles

**Figure 4:**
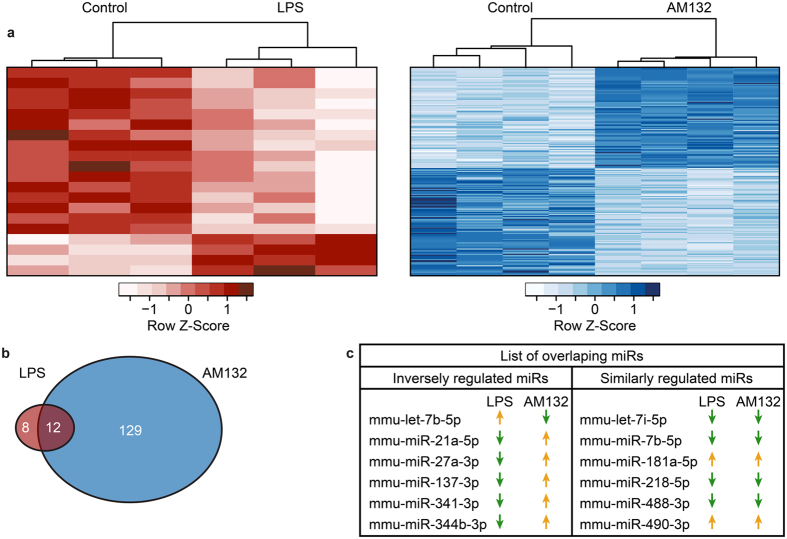
Cortical miR profiling reveals inverse changes under LPS and AM132 treatment. (**a**) Hierarchical clustering dendrograms showing unsupervised clustering and differentially changed miRs under LPS exposure compared with naïve control (Red), and under AM132 treatment compared with random oligonucleotide treated control (Blue). (**b**) Venn diagram showing the number of miRs differentially expressed with LPS/ AM132 and the overlap between them. (**c**) List of miRs overlapping between LPS/AM132 and their changing directions (up or down). Note the largely inverse direction of changes.

To compare the outcome of peripheral miR-132 increases and decreases on the brain and body inflammatory status, we performed cortical miRNA profiling of LPS-induced and AM132 treated samples. RNA-sequencing yielded over 14.5 M reads per cortical sample; with mean 32% alignment to miRBase and 1,199 distinct miRs that were expressed in all samples. In LPS-exposed mice compared to controls, expression analysis using DeSeq2 revealed a small number of 20 differentially expressed cortical miRs (see Supplementary Table S2 for detailed lists of modified brain miRs) (Fig. 4a, n = 3). Although AM132 cannot pass the blood-brain barrier^34^,^35^, peripheral AM132 injection induced differential expression of 129 cortical miRs compared to control brains (Fig. 4b. n = 4, FDR < 0.05, >30 CPM expression; see Supplementary Table S3 for detailed lists of modified brain miRs and Supplementary Fig. S3 for qPCR results of predicted AChE targeting miRs). In both analyses, unsupervised classification correctly separated experimental from control cortices (PCA plot, Supplementary Fig. S3). In spite of the relatively high inter-animal variability in frontal cortex-expressed miRs from LPS-exposed mice, 12 out of the 20 miRs that were differentially expressed between LPS-injected mice and their controls were also differentially expressed in between AM132-injected mice and their controls (Fig. 4b). Six of those 12 miRs (mmu-miR-21a-5p, -27a-3p, -137-3p, -341-3p, -344b-5p and -let-7b-5p) were inversely regulated between AM132 and LPS treatments, and the other six (mmu-miR-7b-5p, -181a-5p, -218-5p, -488-3 and -490-3p) were regulated in the same direction (either up or down) under both treatments (Fig. 4c). Thus, AM132 exposure induced profound and distinct cortical miR changes compared to LPS exposure.

**Figure 5:**
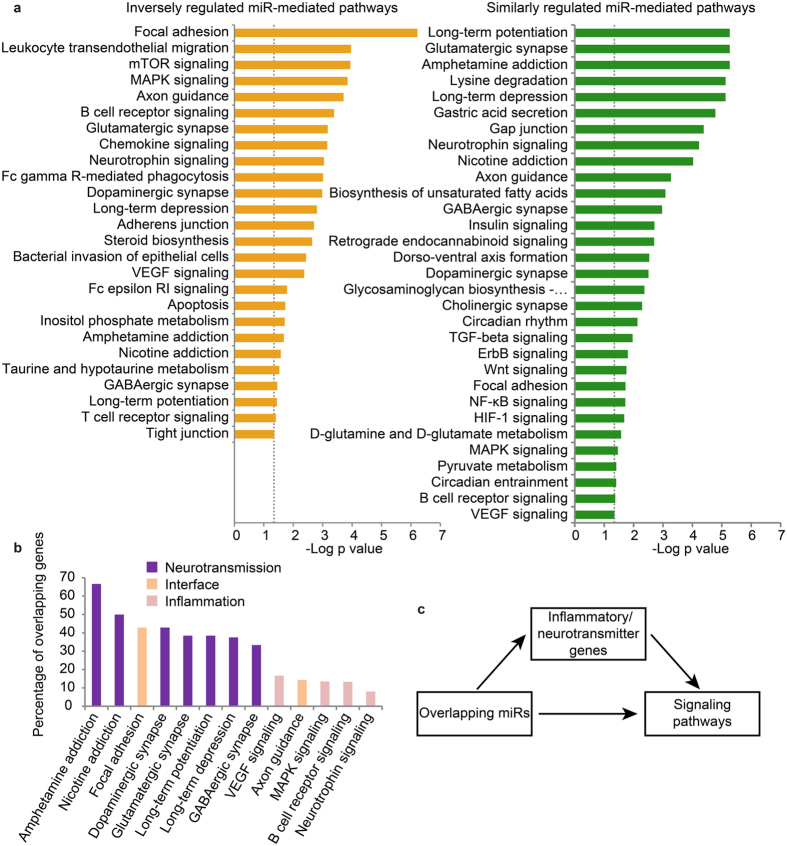
Pathway enrichment analysis of overlapping miRs. (**a**) Inversely and similarly regulated miR-related enriched pathways arranged by the significance of change. (**b**) Overlapping pathways clustered by percent of overlapping miRs in each. Note: neurotransmitter signaling pathways and inflammatory pathways cluster together. (**c**) Scheme of mediation analysis linking miR and pathway changes with modified target genes mediating these pathways.

Next, we ran a miRpath v. 3.0-based pathway analysis (using tarbase v.7 validated target database) updated to current annotations. Specifically, we searched for overlapping miRs and their regulated pathways between the LPS-exposed and AM132-treated groups, with p < 0.05 after FDR correction on both sets (Fig. 5a). This analysis revealed unique enrichment of upregulated cholinergic transcripts under both LPS and AM132, suggesting a potentiated cholinergic response to both of these perturbations. This was accompanied by an overall enrichment for neuronal pathways such as synaptic transmission, but also inflammatory signaling pathways in both groups. Transcripts in 13 of these co-enriched pathways (after filtering 5 pathways relating to systems that do not operate in the brain), could either be up-regulated and/or down-regulated under LPS or AM132. Thus, both AM132 and LPS induced bi-directionally regulated cellular and metabolic pathways that are involved in several neuro-signaling and inflammatory processes, which include dopaminergic, glutamatergic, and GABAergic pathways^16^,^17^.

The modified brain miRs are each targeted to many different coding transcripts. Therefore, we next questioned how many of those targets are shared between the brain pathways that are regulated by both LPS and AM132. Re-clustering of these pathways according to the percent overlap of their validated miR targets (Fig. 5b,c) pointed at both inflammation-controlling and neurotransmission-related targets, such as VEGF, axon guidance, MAPK and neurotrophin receptor as modified under both AM132 and LPS. The neurotransmission controllers were modified more often than inflammation-related ones (binomial test, p < 0.05). To exclude the possibility that size variations in the number of genes per pathway skewed the analysis, we applied a permutation test to miR targets composing these different pathways. This test re-affirmed that brain-modified miRs which change under both AM132 and LPS are enriched for neurotransmitter signaling and inflammatory genes (n = 10,000 permutations, p value < 0.05 for each test). Repeating this permutation test after removal of inflammation and neuronal signaling genes from the analyzed lists deleted the significant difference (p > 0.05) between LPS and AM132-regulated pathways, validating these subgroups as pivotal to the observed differences. Taken together, these findings indicate that LPS and AM132 induced partially similar miR changes involved in both inflammation and neurotransmission, but the LPS-induced changes promoted inflammation whereas AM132-mediated cortical changes suppressed it.

### AM132 co-increases the nAChR and AChE proteins in diaphragm NMJs

**Figure 6:**
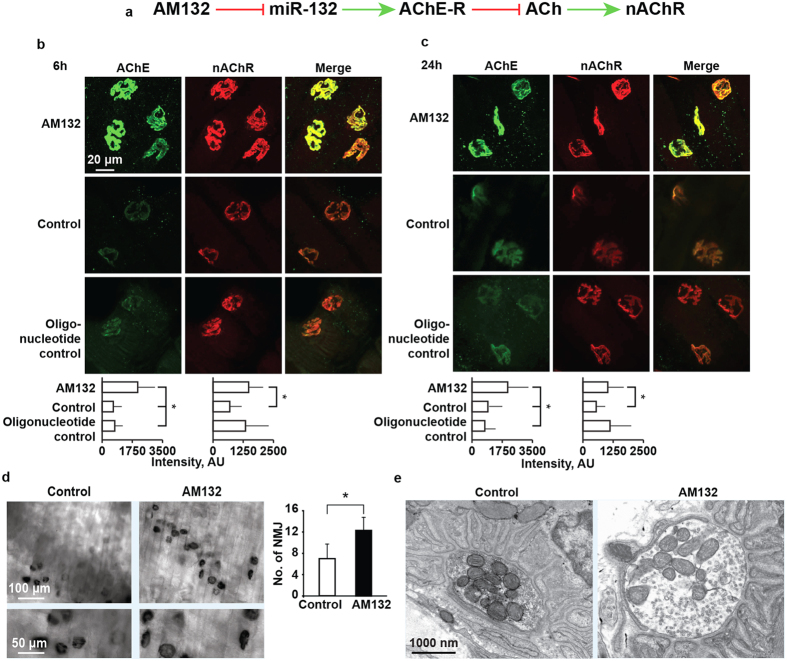
AM132 enhances peripheral cholinergic transmission. **(a**) Schematic presentation of the working hypothesis, where AM132 functionally masks miR-132 inhibition, leading to AChE-R over-expression, blockade of ACh and consequent increase of nAChR. (**b**,**c**) Double staining of AChE and nAChR in diaphragm neuromuscular junctions (NMJs) 6 h and 24 h after IV injection of 3.3 mg/kg AM132, control oligo or PBS. Note AM132-induced enhancement of NMJs nAChR and AChE levels as detected by immunohistochemistry and α-bungarotoxin binding. (**a**) Histochemical activity by Karnovsky’s staining for AChE shows more stained NMJ/Field following 3.3 mg/kg AM132 IV injection. (**e**) Representative TEM micrograph showing characteristic diaphragm NMJ ultrastructure following AM132 injection. One out of 20 electron micrographs, all comparable.

One tissue where blocking miR-132 by AM132 may exert a significant impact is the diaphragm, where balanced response of neuromuscular junctions (NMJs) to cholinergic signals is essential for breathing. Specifically, AM132 would decrease miR-132 levels, elevating the soluble AChE-R protein more than the membrane-bound AChE-S variant. This may functionally diminish the response of nicotinic acetylcholine receptors (nAChR) in NMJs to ACh signals (Fig. 6a). However, the mild Rotarod effect (see Fig. 2g above) indicated that this outcome was minimized, indicating a compensating process. To interrogate the effect of AM132 on the NMJs cholinergic tone, we used preparations of whole diaphragm muscle, known to be extensively innervated by the vagus nerve. Diaphragms were extracted from mice injected with AM132, scrambled (SCR) oligonucleotide control or phosphate-buffered saline (PBS), 6 h and 24 h post-injection. They were then labeled with antibodies directed to AChE or with fluorescein labeled α-Bungarotoxin (αBTX) for tagging the NMJ’s post-synaptic α subunit of the nicotinic ACh receptors (nAChR)^44^. AM132, but not the control oligonucleotide enhanced both AChE and αBTX staining of NMJs at 6 h (ANOVA p = 7.38E-13; n = 105 fields, and p = 0.00091; n = 67 fields, 3 mice/group) and 24 h post-injection (ANOVA p = 0.38E-13; n = 105 fields, and p = 0.00256; n = 71 fields, 3 mice/group), most likely reflecting membrane-bound AChE-S and nAChR. Of note, nAChR is not a target of miR-132; therefore, that AM132 treatment induced massive long-lasting increases in nAChR levels at NMJs may indicate an indirect feedback response to the modified muscle cholinergic tone at this time window, which may minimize the damage to muscle functioning. Supporting this notion, NMJs from oligonucleotide control-injected mice showed faintly increased αBTX staining but non-altered AChE compared to NMJs from control mice (Fig. 6b,c).

Increased AChE protein levels upon AM132 treatment could also reflect increased ACh hydrolytic activity. To test this hypothesis, we dissected intact diaphragms 24 h post-treatment, stained them with acetylthiocholine for catalytically active AChE, and quantified labeled NMJ density (Fig. 6d). AM132-treated mice showed 10.9 ± 4.2 compared to 6.3 ± 2.7 enzyme-stained NMJ/field in each diaphragm of control mice (Student’s t-test P < 0.01, n = 3 mice/group), suggesting selectively enhanced ACh hydrolytic activity under AM132, albeit at a considerably more limited level than the muscle AChE-R mRNA changes. There was, however no sign of structural NMJ changes in transmission electron microscopy, where NMJs from AM132 and PBS-treated diaphragms showed typical and indistinguishable healthy characteristics, and demonstrated sustained membrane fold structures and apparently healthy mitochondria in AM132-treated mice (Fig. 6e), validating the selectivity of the observed differences. The impact of AM132 suppression of AChE-R thus spanned cortical, muscle and circulation effects above and beyond its direct targets.

## Discussion

Injecting the brain-impenetrable AM132 oligonucleotide reduced peripheral miR-132 levels, causing massive, splice variant-specific AChE-R elevation in peripheral tissues while modifying the brain’s miR-132 targets and signaling pathways and suppressing both brain and body inflammation, together reflecting reciprocal body-brain signals that induced more substantial cortical changes in brain miRs and their target transcripts than LPS exposure. Bioinformatics interrogation identified AM132-modified neurotransmission and immune-related circuits culminating with interrupted cholinergic signaling and suppressed pro-inflammatory cytokine transcripts. Elevated AChE-R in brain, serum, and muscle was accompanied by re-balanced cholinergic tone in NMJs, where co-elevated nAChR could expedite the cholinergic response that was weakened by AChE-R elevation. Other examples of splice variant differences that modify the protein product location and properties include the 3′-longer alternative splicing variants of the brain-derived neurotrophic factor (BDNF) and calmodulin-activated protein kinase 2 (Camk2a), which like AChE-R present modified cellular location^45^,^46^,^47^. By immune-labeling neuromuscular junctions, we found AM132 co-elevation of the nicotinic acetylcholine receptor and AChE, re-balancing neurotransmission; which led to mild muscle incoordination. That the peripheral suppression of miR-132 globally reduces inflammation suggests a surprising role of miR-132 as an AChE splice variant-specific bidirectional body-brain regulator.

Our SPR measurement demonstrated 3-fold larger effectiveness of AChE-R suppression by AM132 compared to AChE-S, yet the *in vivo* AM132-mediated difference between the increase in muscle AChE-R and AChE-S was much higher than the SPR measured difference. The SPR measurement however was performed in a synthetic buffer, devoid of the additional molecular components that exist *in vivo*. This context-dependent difference and the apparent increases in the SC35 splice factor and Sirt1 may together explain this contradistinction. Notably, LPS-induced inflammation signals reach the brain, induce ‘sickness behavior’^13^,^16^,^17^,^40^, and change the cholinergic status^48^. In contrast, oligonucleotides are too large to cross the blood-brain barrier^34^,^35^. Nevertheless, unbiased cortical RNA-sequencing revealed larger and inverse impact on cortical miR profiles in AM132-treated as compared to differentially expressed cortical miRs in LPS-exposed mice. This is compatible with miR genes being small and rapidly reacting^15^, and functioning as multi-target dimmers of entire pathways^8^, which may lead to anti-inflammatory regulation by sending smaller molecules to the brain (e.g. ACh).

MiR-132 knockout induces modest brain-related functional phenotypes^49^, but the global cortical reactions to partial and temporary changes in miR-132 levels remained largely unknown. To the best of our knowledge, this is the first example of a differential effect of a miR on specific splice variants and the consequent changes in its validated targets and their downstream neuro-modulatory and inflammatory cytokines. Specifically, the AChE-S transcript was significantly decreased under LPS exposure, indicating limited synaptic ACh hydrolysis and therefore exacerbated cholinergic signaling and increased anti-inflammatory protection; in contrast, cortical AChE-S was increased under AM132, possibly reflecting peripheral body-brain cholinergic messages known to accompany stress insults^31^. That the stress-induced soluble AChE-R variant^7^,^50^ was significantly increased both under LPS and following AM132 may possibly reflect general differences in alternative splicing and increases in the splicing factor SC35 in these systems^41^. At the biochemical level, elevated AChE-S activity may suppress the stress-characteristic hyper-activation of synaptic neurotransmission^26^, whereas AChE-R elevation might reduce the cholinergic blockade over brain NFkB. A parallel bidirectional change was also observed in NMJs (where both nAChR and AChE were increased), suggesting inversely directed checks and balances aimed to achieve homeostasis.

Other quantified miR-132 target transcripts include the transcriptional co-activator P300-CBP^22^, growth factor HB-EGF, and SIRT1. P300-CBP controls both miR-132 expression and innate immune response to viral infection. HB-EGF regulates the NFkB pathway in a context- dependent manner^23^, and may thus interfere with cytokine production^51^. In cellular injury, miR-132 is upregulated and HB-EGF decreases, reducing leukocytes capability to attract chemokines and divert the healing process from inflammation towards proliferation^52^. Similarly, miR-132 levels increased and HB-EGF decreased under LPS, but AM132 yielded the opposite outcome, mimicking healing conditions. SIRT-1 is another more upstream epigenetic modulator^53^; its decrease is associated with both liver^25^ and brain^54^ inflammation^53^, impaired energy metabolism^25^, and ageing^54^. SIRT-1 showed LPS-induced decreases and AM132-induced increases, which might protect from inflammation^55^. Several cytokines, including IL-6, IL-18, IL-1β, TNFα and MCP1 were also increased under LPS and decreased under AM132. This observation is compatible with the cortical decline of anti-inflammatory miRs under LPS and their increase under AM132.

The profound impact of AM132 on miRNA and mRNA cortical transcripts likely reflects the hierarchically high position of miR-132 and its downstream targets as regulators of cortical gene expression. Examples of the AM132-modulated miRs include let-7b, which regulates miR metabolism pathways^56^ and was up-regulated under LPS and suppressed in AM132-treated brains, supporting the recently reported functional link between miR regulation of anxiety and metabolism^57^. Also, miR-21 and miR-27a are both suppressors of inflammation^56^ that were down-regulated under LPS and up-regulated by AM132, compatible with the exacerbated and suppressed inflammation in these two conditions. Overlapping pathways that came up as enriched for differentially expressed miRs between AM132 and LPS treatments included synaptic transmission and inflammatory signaling. We found inflammatory signaling pathways to be primarily affected by inversely expressed miRs between LPS and AM132 treatments. However, despite the limited LPS-mediated changes in cortical miR expression profiles, miRs targeting pathways of the cholinergic system were similarly regulated in both the LPS and AM132 treatment groups. Further studies will be required to find out if these distinctions reflect cholinergic-related compensation processes aimed to re-achieve homeostasis, and if this occurs under diverse perturbations.

AM132 cannot cross the blood-brain barrier^35^, which implies that its brain impact mainly reflects the outcome of its peripheral body-to-brain signals. For example, by targeting peripheral AChE^26^,^27^, miR-132 might elevate ACh levels and activate efferent vagal messages. AM132 would reduce miR-132 levels, possibly limiting exosomal transport of miR-132 to the brain. Although AM132 treatment caused no change in brain miR-132 levels, it led to upregulated AChE expression and hydrolytic activity in both brain and muscle. Immunohistochemistry and histo-chemical staining further confirmed elevated AChE labeling in otherwise healthy-looking NMJs from AM132-treated mice within the short 24 hours span of our tests. The numbers of NMJs stained for AChE were increased, perhaps reflecting somewhat more densely packed AChE-S in these synapses; and nAChRs was likewise upregulated in NMJs, possibly balancing the massive increase in muscle AChE-R, which competes with NMJ activation. We also identified a mild AM132-induced fatigue, i.e. mice falling off earlier in the third trial of Rotarod tests, but no overt changes were observed in TEM muscle morphology and NMJ structures. The imbalanced nAChR/AChE state might hence explain this mild muscular weakness^58^. In conclusion, we report inverse cortical response to LPS and the inhibition of miR-132 in the periphery, pointing to a body-to-brain anti-inflammatory que that was reciprocal to miR-132 induction by peripheral LPS. Together, that AM132 treatment not only affected peripheral tissues such as NMJs, but also the brain, underlines the high- hierarchy regulation by miR-132 in the context of neuro-inflammation^13^,^16^,^17^, and lead to the identification of selectivity of miR-132 to AChE-R over AChE -S.

## Methods

### Experimental design

#### Design and synthesis of AM132

The 2′-O-methyl antisense oligonucleotides were prepared on an Expedite 8909 Nucleic Acid Synthesizer (Applied Biosystems) on a phosphorothioate backbone, where the non-bridging oxygen was replaced with a sulfur atom. The AM132 Sequence was: 5′ CGA CCA UGA CUG UAG ACU GUU A 3′. A scrambled oligonucleotide with no apparent match in the mouse genome was composed of a mixture of the same bases (Supplementary Fig. S1).

#### Ethics Statement

All methods were carried out in accordance with the Animal Care and Use Guidelines of the Hebrew University (NS-10-12745-5 for injection experiments) which follow the National Research Council (US) Guide for Care and Use of Laboratory Animals. All experimental protocols were approved by the University Ethics Committee for Maintenance and Experimentation on Laboratory Animals, The Hebrew University, Jerusalem, Israel.

#### Animals

Male 4 months old Balb/C mice were intravenously (i.v.) injected with 3.3 mg/kg AM132 or a scrambled oligonucleotide or PBS as controls. After 24 hours all mice were euthanized and their brains, gastrocnemius muscle, diaphragm muscle and serum were collected and used for the following experiments: RNA extraction, small RNA-sequencing, ACh hydrolytic AChE activity tests, electron microscopy and qRT-PCR measurements. Protein content was measured by the Lowry assay. LPS-induced inflammation experiments were as described before^48^ and frontal cortex RNA was used for miRs profiling, and for quantifying mRNA transcript levels.

#### Surface plasmon resonance

Surface plasmon resonance experiments^59^ were conducted using a Biacore 3000 instrument (Biacore AB, Uppsala, Sweden). Standard buffer HBS (10 mM HEPES, 150 mM NaCl, 3.4 mM EDTA, 0.005% surfactant P20, pH 7.4) was used for the analyses carried out at 25 °C. Biotinylated oligonucleotides were dissolved in 100% 1,1,1,3,3,3- hexafluoro-2-propanol to 1 mM, diluted (1:5000) into 10 mM sodium acetate pH 5.0 and injected at 10 ml/min. The AChE-R and AChE-S, SIRT1, and miR-132 binding site oligonucleotides were diluted in buffer (0.3125, 0.625, 1.25, 2.5, 5 and 10 mM), captured on the chip or injected over the flow cells for 2 min at 10 ml/min, with 5-min association and 5-min dissociation, except for the highest concentration that was allowed to dissociate for 1 h. The sensograms were double-referenced and were fit using a mathematical model of a simple 1:1 interaction (Scrubber 2 soft- ware).

#### RNA extraction

RNA extraction from tissue samples was carried out using miRNeasy (Qiagen, Valencia, CA, USA) as per the manufacturer’s instructions. DNAse treatment was applied, RNA concentration and integrity confirmed by Nanodrop and gel electrophoresis respectively.

#### Sequencing data analysis and normalization

##### Construction of small RNA libraries and Sequencing

Construction of libraries was done using the NEB-Next multiplex Small RNA Preparation Kit (New England BioLabs, USA) according to the manufacturer’s instructions using 150 ng RNA per sample. Next, individual libraries were adjusted to 2 nM and pooled before denaturation and dilution according to Illumina’s instructions. cDNA libraries were prepared from gel-extracted size-selected RNA with a median length of 140 nucleotides. Library quality for miRs was determined by TapeStation and BioAnalyzer. APhiX control library was spiked in, to determine the sequencing error rate (<1%). The diluted libraries (20 pM) were loaded onto a 4-lane flow cell for cluster formation using the TruSeq™ SR Cluster Kit v3 (Illumina). Sequencing was performed on an Illumina NextSeq 500 system. Libraries were sequenced from the 5′- end using a read length of 30 nt and results converted into fastq files with Casava 1.8.2 (Illumina) using BaseSpace software and downloaded as Fastq files for alignment and further analysis.

##### Alignment and normalization

Raw sequence counts were processed in a bioinformatics pipeline including a quality control check (FastQC), adapter trimming, consolidation of reads across samples, and alignment of reads against a reference database (miRBase v. 21) using miRExpress 2.0. Sequences were normalized using the RLE method (DeSeq2) as well as by assessing counts-per-million (CPM) values to correct for library size. Quality check for genomic homology across groups was analyzed by PCA binomial plot and unbiased hierarchical clustering (Supplementary Fig. S3).

##### Expression analysis and statistics

Differential expression was analyzed using DeSeq2^60^ using likelihood ratio tests for both LPS and AM132 datasets. Differences were deemed significant for p < 0.05 after FDR correction and filtered for a mean greater than 15 CPM across all samples. Additionally, a non-parametric permutation test (10,000 permutations) was run independently for all pairwise samples in each group. Differentially expressed miRs of the LPS and AM132 groups were analyzed.

##### Pathway enrichment Analysis

Pathway enrichment analysis was done using miRpath v. 3.0 (tarbase v.7)^61^. We identified overlapping miRs and enriched pathways between the LPS-exposed and AM132-treated groups, with p < 0.05 after FDR correction. Similarly and inversely regulated overlapping pathways targeted by the differentially expressed miRs were clustered via an unsupervised procedure using the fraction of overlapping genes in these pathways (number of genes shared in a pathway between inversely and similarly regulated miR groups divided by the supremum analysis of miR targets in the pathway). The clustered pathways were then deemed to significantly separate between inflammatory and signaling genes using a two-way binomial test. Mediation type analysis was done using permutation tests (n = 10,000) for inflammatory and neurotransmission signaling targets.

### qRT PCR

RNA samples were used for synthesis of cDNA, using Quanta cDNA synthesis kit for mRNA and qScript microRNA cDNA Synthesis Kit for miRs as per manufacturer’s (Quanta Biosciences) protocol. Quantitative real time- PCR (qRT-PCR) was performed using ABI prism 7900 HT and SYBR green master mix (Quanta Biosciences) with or without ROX for mRNA and miR transcripts, respectively. The mRNA primer sequences are detailed in Supplementary Table S4. Serial dilution of samples was used to evaluate primers efficiency. Results were normalized to the expression level of actin. For miRs, PerfeCTa microRNA assay primers (Quanta Biosciences) were used. Results were normalized to the expression of snoRD47. Fold change values for both miRs and mRNAs were calculated using the ΔΔCt method.

### AChE activity

AChE activity in serum, brain and gastrocnemius muscle was determined by microtiter plate assay by measuring ATCh (1 mmol/L; Sigma-Aldrich, St. Louis, MO, USA) hydrolysis rate^62^ after pre-incubation with a specific BChE inhibitor (iso-OMPA (Sigma-Aldrich), in the dark at a concentration of 500 μmol/L. Enzyme activities were calculated using 405 nm absorbance value for 5-thio-2- nitrobenzoate produced from dithionitrobenzoate reaction with the hydrolysis product, thiocholine (13,600 [mol·L]/cm). The non-enzymatic breakdown of substrate was subtracted from the total rate of hydrolysis.

### Rotarod test for measuring muscle fatigue

An accelerating Rotarod (Ugo Basile, Comerio VA, Italy) was used for testing muscle fatigue. The Rotarod was set to accelerate from 4 to 40 rpm over 5 min. Mice were then tested for 3 successive times with a minimum of 2 min elapsed between trials.

### Immunocytochemistry and α-bungarotoxin double staining

Diaphragms were isolated as described before^63^ and were fixed in 4% paraformaldehyde in PBS for 1 h., permeabilized in 0.1%Triton X-100 for 20 min, blocked in 5% horse serum for 4 h., and then incubated with primary antibody anti-AChE (1:200, Santa Cruz, CA, USA) overnight at 4 °C. Cy2-conjugated secondary antibodies were then added for 4 h at R.T. During the last wash 10 nM α-bungarotoxin-Cy3 conjugate (Sigma, St. Louis, MO, USA) was added for 30 min to mark α-subunits of the neuromuscular nicotinic receptors. Coverslips were rinsed twice with PBS and once with DDW and were mounted for electron microscopy (FluoroMount-G, Electron Microscopy Sciences, Washington, PA, USA). Slides were scanned using the Olympus FV-1000 confocal microscope as above. Image Analysis was performed using the ImageJ-software (National Institutes of Health, Maryland, USA).

### Histochemical AChE activity staining of diaphragms

AChE activity can be visualized in situ by staining with acetylthiocholine in the presence of copper and ferrous ions, which together form a brown precipitate^64^. The mice were sacrificed and dissected to expose the diaphragm muscle. After two washes with PBS, the diaphragms were incubated in Karnovsky’s staining solution including acetylthiocholine at R.T. Within 20 min, we could visualize NMJ numbers, size and AChE activity. AChE staining intensity for each NMJ was measured by applying a visual scale of 1-5 for 15 different fields of 100 um square each per diaphragm, and average ± SD intensity values were calculated for each diaphragm. The number of NMJs, their size and the intensity of staining were compared.

### Electron Microscopy

Diaphragms were removed with the surrounding ribs to prevent folding, washed twice with PBS and were fixed with 2.5% glutaraldehyde and 2% formaldehyde in 0.1 M cacodylate buffer (containing 1% OsO4 and 1.5% potassium ferrycyanide) solution, pH 7.4, 2 h at R.T., dehydrated in a graded ethyl alcohol series followed by propylene oxide, and embedded in Agar 100 resin. Polymerization was carried out at 60 °C for 48 h. Ultrathin sections were cut on an LKB III ultratome, mounted on copper 200 mesh thin bar grids and stained with uranyl acetate and lead citrate. Pictures were captured on a Tecnai-12 electron microscope (Phillips, The Netherlands) equipped with Megaview II CCD camera and 3.0 analysis software (Soft Imaging System, GmbH, Germany).

### Statistical analysis

Ellman’s assay and qRT PCR results were analyzed by T-test followed by Tukey-test and compared with controls. Microscopy and behavior tests were analyzed by ANOVA. Sequencing statistics analysis details are mentioned in the corresponding Methods section. Results are expressed as mean ± S.E.M, with p < 0.05 considered statistically significant.

## Additional Information

**Accession codes:** All of the sequencing data has been uploaded to Gene Expression Omnibus under accession code SUB1926319.

**How to cite this article:** Mishra, N. *et al*. Antisense miR-132 blockade via the AChE-R splice variant mitigates cortical inflammation. *Sci. Rep.* **7**, 42755; doi: 10.1038/srep42755 (2017).

**Publisher's note:** Springer Nature remains neutral with regard to jurisdictional claims in published maps and institutional affiliations.

## Supplementary Material

Supplementary Information
