# Supplementary material for: Antisense miR-132 blockade via the AChE-R splice variant mitigates cortical inflammation
Source: Sci Rep. 2017 Feb 17;7:42755. doi: 10.1038/srep42755 (PMC5314396; doi:10.1038/srep42755)
Supplement: Supplementary Information [file srep42755-s1.pdf]

Supplementary Material for:

Antisense miR-132 blockade via the AChE-R splice variant mitigates cortical inflammation

Nibha Mishra,[1,2¥] Lyndon Friedson,[1,2¥] Geula Hanin,[1,2] Uriya Bekenstein,[1,2] Meshi Volovich,[1], Estelle R. Bennett[1,2]
David S. Greenberg[1,2] and Hermona Soreq[1,2,*]
[1]The Edmond and Lily Safra Center for Brain Sciences, and [2]The Silberman Institute of Life Sciences, The Hebrew University of Jerusalem, The Edmond Safra Campus, Givat Ram, Jerusalem 9190401, Israel.

Supplementary figures

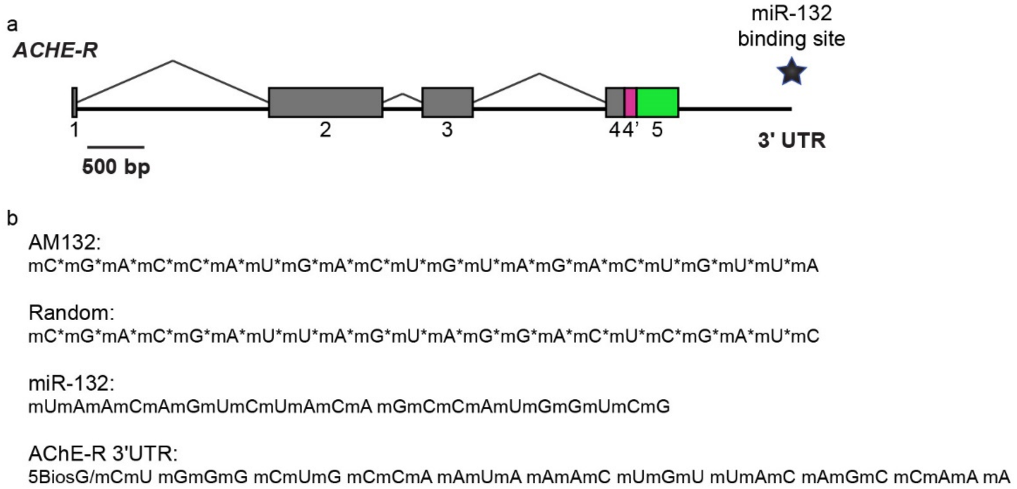

Supplementary Figure 1: The employed sequences. Shown are the AM132 and random oligonucleotides that were employed in this study, as well as the synthetic miR-132 and complementary 3'-UTR sequences that were tested by SPR measurements (m=methyl group; *on the sequence notes phosphorothioate protection; starred structure notes the binding site location.

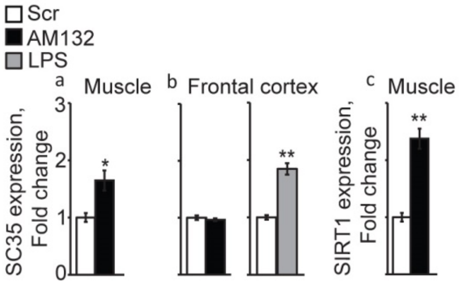

Supplementary Figure 2: AM132 increases muscle, but not brain levels of both the stress-induced splicing factor SC35 and the epigenetic regulator Sirt1, whereas LPS elevates SC35 in the brain. Following AM132 treatment, an in increase in SC35 expression was observed in (a) muscle with AM132 treatment and (b) brain with LPS exposure, but no change in brain with AM132 treatment. Note these changes are in congruence with changes in miR-132 in these systems. (c) Sirt1 expression increased with AM132 treatment as found through SPR analysis.

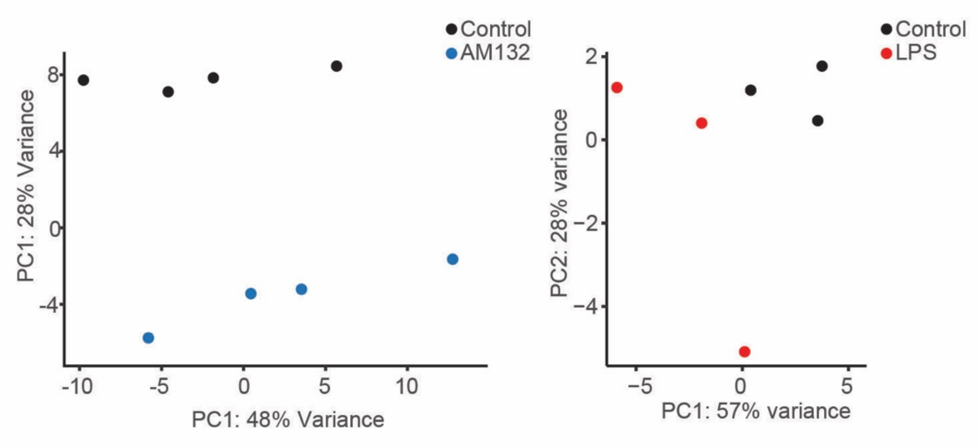

Supplementary Figure 3: Segregation of miR profile samples as per group. Shown is PCA analysis of all of the miRs identified as differentially expressed in cortical tissues from mice belonging to the LPS and AM132 treated groups. Note that both AM132 treated and LPS-exposed samples were well segregated from their respective controls, with AM132 segregation resolution being better than LPS treatment group.

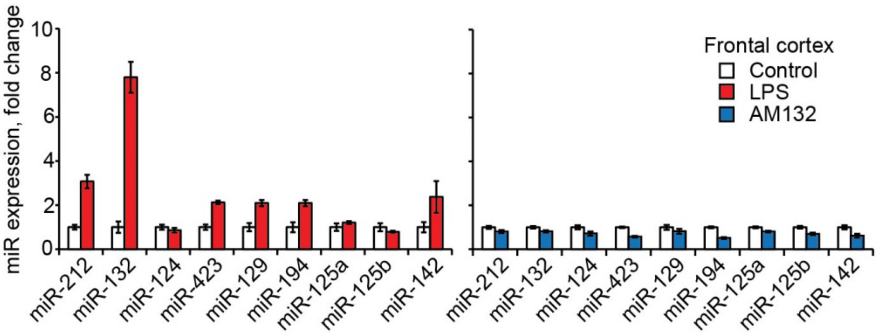

Supplementary Figure 4: CholinomiRs expression in the frontal cortex. TargetScan predicted miRs targeting cholinergic transcripts were largely upregulated following LPS exposure but were not changed under AM132 treatment, suggesting LPS-induced suppression of stress and inflammation-inducible cholinergic signaling and inverse AM132-mediated potentiation of cholinergic reactions in the brain.

Supplementary Tables

Supplementary Table 1: Sequences used in surface plasmon resonance analysis

Name	Sequence	mer	Modifications	Purity
miR132	mUmAmAmCmAmGmUmCmUmAmCmA mGmCmCmAmUmGmGmUmCmG	22	Fully phosphorothioated and 2'-o-methylated	HPLC
Biotin-AChE-S 3'UTR/miR-132	/5BiosG/mCmU mGmGmG mCmCmG mCmCmA mAmUmA mAmAmC mUmGmU mUmAmC mAmGmC mCmAmC mC	30	Fully phosphoropthioated and 2'-o-methylated	HPLC
Biotin-AChE-R 3'UTR/miR-132	/5BiosG/mCmU mGmGmG mCmUmG mCmCmA mAmUmA mAmAmC mUmGmU mUmAmC mAmGmC mCmAmA mA	30		HPLC

			Fully phosphorothioated and 2'-o-methylated	
Biotin-miR-132/SIRT1	/5BiosG/mAmC mUmGmG mCmAmU mAmUmG mUmUmU mUmGmU mAmGmA mCmUmG mUmUmU mAmAmU mG	30	Fully phosphorothioated and 2'-o-methylated	HPLC

See Hanin et al., 2014 for further details on the composition and purity level of these oligonucleotides.

Supplementary Table 2: The expression levels of 20 differentially expressed miRNAs in the comparison of LPS to controls

	Base mean	Log_2 fold change	Log_2 fold change-Standard error	Statistic	P value	P adjusted
mmu-miR-21a-5p	42891.86	-1.03	0.23	-4.49	7.28E-06	0.002
mmu-miR-181a-5p	105808.60	0.49	0.13	3.79	1.50E-04	0.005
mmu-miR-218-5p	31137.49	-0.67	0.17	-3.87	1.11E-04	0.005
mmu-miR-27a-3p	6267.31	-0.65	0.17	-3.83	1.26E-04	0.005
mmu-miR-7b-5p	12919.41	-0.67	0.17	-3.85	1.17E-04	0.005
mmu-miR-344b-3p	1002.94	-0.71	0.18	-3.98	6.89E-05	0.005
mmu-miR-490-3p	1220.58	0.61	0.17	3.51	4.48E-04	0.013
mmu-miR-488-3p	1273.73	-0.65	0.20	-3.31	9.47E-04	0.024
mmu-miR-341-3p	15028.66	-0.42	0.13	-3.17	1.51E-03	0.028
mmu-miR-9-5p	435022.89	-0.61	0.19	-3.19	1.43E-03	0.028
mmu-miR-1839-5p	5806.74	-0.54	0.17	-3.21	1.30E-03	0.028
mmu-miR-146a-5p	2161.21	-0.79	0.26	-3.07	2.14E-03	0.034
mmu-let-7i-5p	83108.77	-0.58	0.19	-3.08	2.08E-03	0.034
mmu-let-7g-5p	138693.25	-0.56	0.18	-3.04	2.38E-03	0.035
mmu-miR-129-1-3p	2413.03	0.44	0.15	3.02	2.54E-03	0.035
mmu-miR-379-5p	15429.70	-0.52	0.18	-2.92	3.53E-03	0.045
mmu-let-7b-5p	34600.74	0.42	0.14	2.89	3.87E-03	0.047
mmu-miR-369-3p	987.12	-0.66	0.23	-2.87	4.12E-03	0.047
mmu-miR-137-3p	1085.00	-0.52	0.18	-2.82	4.74E-03	0.049
mmu-miR-376b-5p	865.93	-0.55	0.19	-2.83	4.60E-03	0.049

Supplementary Table 3: The expression levels of 141 differentially expressed miRNAs in the comparison of AM132 to controls

	Base mean	Log_2 fold change	Log_2 fold change-Standard error	Statistic	P value	P adjusted
mmu-miR-22-3p	58215.84	2.25	0.12	19.47	1.97E-84	5.78E-82
mmu-miR-146a-5p	2119.86	-2.12	0.14	-14.70	6.54E-49	9.58E-47
mmu-miR-30e-5p	48559.67	1.49	0.12	12.78	2.11E-37	2.06E-35
mmu-miR-127-3p	52309.84	2.17	0.18	12.37	3.67E-35	2.69E-33
mmu-let-7i-3p	365.07	1.95	0.19	10.09	5.96E-24	3.49E-22
mmu-miR-151-5p	4845.47	-2.68	0.28	-9.67	4.24E-22	2.07E-20
mmu-miR-30e-3p	10181.02	-1.16	0.12	-9.34	9.52E-21	3.99E-19

mmu-miR-3535	496.84	1.57	0.17	9.10	8.97E-20	3.29E-18
mmu-miR-30a-3p	9413.88	-1.03	0.12	-8.33	8.10E-17	2.64E-15
mmu-miR-423-3p	3770.88	1.03	0.13	7.94	2.01E-15	5.90E-14
mmu-miR-879-5p	343.06	1.46	0.18	7.92	2.46E-15	6.56E-14
mmu-miR-136-3p	25900.16	1.34	0.17	7.84	4.47E-15	1.09E-13
mmu-miR-872-3p	406.54	1.41	0.18	7.76	8.81E-15	1.99E-13
mmu-miR-488-3p	4012.71	-0.99	0.13	-7.74	9.70E-15	2.03E-13
mmu-miR-379-3p	1318.29	-1.21	0.16	-7.33	2.26E-13	4.41E-12
mmu-miR-374b-5p	1542.47	-1.02	0.15	-7.01	2.35E-12	4.31E-11
mmu-miR-340-5p	37409.30	1.06	0.15	6.83	8.35E-12	1.44E-10
mmu-miR-106b-3p	977.07	-0.98	0.14	-6.80	1.01E-11	1.65E-10
mmu-miR-140-5p	2643.65	0.89	0.13	6.76	1.36E-11	2.10E-10
mmu-miR-219a-5p	607.93	1.86	0.28	6.64	3.20E-11	4.69E-10
mmu-miR-873a-5p	2835.26	1.00	0.15	6.62	3.54E-11	4.94E-10
mmu-miR-29c-3p	1422.93	1.15	0.18	6.56	5.54E-11	7.38E-10
mmu-miR-126a-5p	3751.25	1.08	0.17	6.42	1.39E-10	1.77E-09
mmu-miR-25-3p	4906.95	-1.17	0.18	-6.35	2.13E-10	2.60E-09
mmu-miR-377-3p	339.83	1.24	0.20	6.25	4.01E-10	4.70E-09
mmu-miR-144-3p	1044.38	1.88	0.30	6.21	5.30E-10	5.97E-09
mmu-miR-485-3p	4667.32	-1.24	0.20	-6.16	7.49E-10	8.12E-09
mmu-miR-666-5p	782.19	-1.34	0.22	-6.08	1.20E-09	1.25E-08
mmu-miR-344-3p	12888.21	1.34	0.22	6.04	1.55E-09	1.51E-08
mmu-miR-151-3p	14019.12	-1.13	0.19	-6.04	1.54E-09	1.51E-08
mmu-miR-181c-3p	771.27	1.08	0.18	5.98	2.29E-09	2.10E-08
mmu-miR-672-5p	377.38	-1.09	0.18	-5.98	2.29E-09	2.10E-08
mmu-miR-708-5p	3819.96	0.72	0.12	5.79	7.03E-09	6.24E-08
mmu-let-7i-5p	222434.22	-0.73	0.13	-5.78	7.60E-09	6.55E-08
mmu-miR-504-5p	367.00	-1.04	0.18	-5.73	9.81E-09	8.21E-08
mmu-miR-129-5p	57754.88	-0.87	0.15	-5.72	1.04E-08	8.48E-08
mmu-let-7c-5p	134465.00	-0.91	0.16	-5.71	1.12E-08	8.84E-08
mmu-miR-381-3p	112683.40	-1.33	0.24	-5.62	1.91E-08	1.47E-07
mmu-miR-181c-5p	1255.10	0.88	0.16	5.41	6.15E-08	4.62E-07
mmu-miR-20a-5p	732.56	0.88	0.16	5.32	1.03E-07	7.56E-07
mmu-miR-431-5p	376.34	-1.30	0.25	-5.23	1.71E-07	1.22E-06
mmu-miR-330-5p	11162.86	-0.95	0.19	-5.10	3.42E-07	2.39E-06
mmu-miR-872-5p	6150.45	0.73	0.15	5.03	4.90E-07	3.34E-06
mmu-miR-148a-3p	89666.50	-1.43	0.29	-4.97	6.66E-07	4.43E-06
mmu-miR-369-5p	4680.61	0.85	0.17	4.89	1.02E-06	6.61E-06
mmu-miR-30b-3p	298.07	-0.79	0.17	-4.76	1.97E-06	1.26E-05
mmu-miR-1843b-3p	2603.96	-0.88	0.19	-4.71	2.44E-06	1.52E-05
mmu-miR-101a-3p	56847.81	0.96	0.20	4.67	3.02E-06	1.84E-05
mmu-miR-93-5p	1564.62	0.61	0.14	4.51	6.63E-06	3.97E-05
mmu-miR-138-5p	8698.63	0.87	0.19	4.49	7.27E-06	4.26E-05
mmu-miR-1298-5p	5309.81	-2.20	0.49	-4.46	8.28E-06	4.76E-05
mmu-miR-140-3p	8698.84	0.78	0.18	4.42	9.82E-06	5.54E-05
mmu-miR-192-5p	3577.06	0.63	0.15	4.33	1.51E-05	8.35E-05

mmu-miR-423-5p	1004.85	-0.59	0.14	-4.32	1.58E-05	8.60E-05
mmu-miR-541-5p	25176.71	0.69	0.16	4.22	2.39E-05	0.00013
mmu-miR-434-3p	41710.58	0.40	0.09	4.22	2.47E-05	0.00013
mmu-let-7a-1-3p	1229.26	0.85	0.20	4.20	2.62E-05	0.00013
mmu-let-7c-2-3p	1228.61	0.85	0.20	4.20	2.62E-05	0.00013
mmu-miR-1198-5p	3457.04	-0.78	0.19	-4.10	4.10E-05	0.00020
mmu-miR-485-5p	4360.76	-0.77	0.19	-3.99	6.48E-05	0.00032
mmu-let-7f-5p	382252.28	-0.63	0.16	-3.96	7.41E-05	0.00036
mmu-miR-21a-5p	58475.35	0.71	0.18	3.96	7.55E-05	0.00036
mmu-miR-92b-3p	4553.62	-0.63	0.16	-3.95	7.74E-05	0.00036
mmu-miR-212-5p	16420.64	0.50	0.13	3.90	9.61E-05	0.00044
mmu-miR-148b-3p	9624.45	-0.61	0.16	-3.88	0.00010	0.00047
mmu-miR-328-3p	51583.27	-0.64	0.17	-3.87	0.00011	0.00048
mmu-miR-185-5p	10751.79	0.49	0.13	3.84	0.00012	0.00053
mmu-miR-10a-5p	345.26	-1.05	0.28	-3.83	0.00013	0.00055
mmu-miR-467a-5p	529.47	-0.96	0.25	-3.82	0.00013	0.00056
mmu-miR-376a-5p	514.75	0.61	0.16	3.82	0.00013	0.00056
mmu-miR-467b-5p	530.08	-0.96	0.25	-3.81	0.00014	0.00057
mmu-miR-181a-1-3p	2265.98	0.70	0.18	3.79	0.00015	0.00063
mmu-miR-874-3p	1431.64	0.58	0.16	3.67	0.00024	0.00097
mmu-miR-410-3p	4714.70	0.65	0.18	3.66	0.00025	0.00098
mmu-miR-27a-3p	11298.81	0.49	0.13	3.66	0.00025	0.00098
mmu-miR-139-5p	43417.03	-0.91	0.25	-3.61	0.00031	0.00119
mmu-let-7e-5p	18172.09	-0.45	0.13	-3.56	0.00036	0.00139
mmu-miR-29a-3p	116786.04	0.42	0.12	3.56	0.00037	0.00140
mmu-miR-187-3p	636.62	-1.77	0.51	-3.50	0.00047	0.00174
mmu-miR-382-3p	6486.78	-0.74	0.21	-3.47	0.00052	0.00191
mmu-miR-99b-3p	1141.44	-0.56	0.16	-3.45	0.00055	0.00200
mmu-miR-137-3p	2961.45	0.72	0.21	3.45	0.00057	0.00203
mmu-miR-341-3p	16628.92	0.47	0.14	3.44	0.00059	0.00207
mmu-let-7b-5p	36479.71	-0.46	0.13	-3.40	0.00067	0.00233
mmu-miR-222-3p	15332.37	0.46	0.14	3.39	0.00070	0.00241
mmu-miR-708-3p	16401.36	-0.66	0.20	-3.36	0.00078	0.00265
mmu-miR-125b-1-3p	1018.40	-0.57	0.17	-3.36	0.00079	0.00265
mmu-miR-135a-5p	3261.26	0.56	0.17	3.35	0.00081	0.00269
mmu-miR-181a-5p	109401.76	0.49	0.15	3.33	0.00087	0.00285
mmu-miR-7a-1-3p	299.57	-0.69	0.21	-3.31	0.00092	0.00301
mmu-miR-431-3p	1042.28	-0.64	0.20	-3.27	0.00106	0.00341
mmu-miR-30c-5p	79793.15	-0.40	0.13	-3.14	0.00169	0.00539
mmu-miR-181b-5p	26126.85	0.41	0.13	3.13	0.00174	0.00547
mmu-miR-409-3p	16907.89	-0.77	0.25	-3.11	0.00187	0.00581
mmu-miR-425-5p	743.82	0.48	0.16	3.08	0.00205	0.00632
mmu-miR-3068-5p	1159.96	0.37	0.12	3.08	0.00209	0.00638
mmu-miR-344b-3p	1656.42	0.62	0.20	3.05	0.00230	0.00694
mmu-miR-1981-5p	2823.71	-0.73	0.24	-2.99	0.00277	0.00829
mmu-miR-128-3p	1927174.90	-0.49	0.17	-2.94	0.00324	0.00958

mmu-miR-106b-5p	294.38	0.51	0.17	2.93	0.00338	0.00991
mmu-miR-150-5p	2742.07	0.67	0.23	2.93	0.00344	0.00999
mmu-miR-31-5p	476.24	0.51	0.18	2.89	0.00386	0.01107
mmu-miR-218-5p	114399.31	-0.66	0.23	-2.88	0.00402	0.01144
mmu-miR-669c-5p	389.02	-0.69	0.24	-2.86	0.00417	0.01153
mmu-miR-145a-3p	1046.57	0.54	0.19	2.87	0.00414	0.01153
mmu-miR-335-3p	6539.55	-0.53	0.19	-2.87	0.00413	0.01153
mmu-miR-203-3p	1631.64	0.52	0.18	2.85	0.00437	0.01196
mmu-miR-144-5p	314.09	0.81	0.28	2.85	0.00443	0.01202
mmu-miR-7a-5p	101123.69	-0.95	0.34	-2.82	0.00485	0.01305
mmu-miR-124-3p	52911.24	0.51	0.18	2.81	0.00498	0.01327
mmu-let-7a-5p	100430.52	-0.57	0.20	-2.80	0.00509	0.01343
mmu-miR-98-5p	5862.22	-0.48	0.17	-2.80	0.00517	0.01353
mmu-miR-1983	740.03	-0.50	0.18	-2.76	0.00570	0.01477
mmu-miR-411-5p	27748.17	0.39	0.14	2.75	0.00588	0.01498
mmu-miR-671-3p	858.28	-0.49	0.18	-2.76	0.00584	0.01498
mmu-miR-195a-3p	471.95	-0.64	0.23	-2.73	0.00638	0.01610
mmu-miR-153-3p	3392.87	-0.60	0.22	-2.67	0.00752	0.01883
mmu-miR-770-5p	660.65	0.71	0.27	2.66	0.00771	0.01914
mmu-miR-344d-3p	3411.26	-0.67	0.25	-2.64	0.00833	0.02050
mmu-miR-339-5p	281.14	0.56	0.21	2.64	0.00841	0.02052
mmu-miR-668-3p	1956.52	-0.38	0.14	-2.60	0.00943	0.02227
mmu-miR-16-1-3p	363.52	0.53	0.20	2.60	0.00937	0.02227
mmu-miR-673-5p	349.71	-0.44	0.17	-2.60	0.00935	0.02227
mmu-miR-6540-5p	639.28	-0.73	0.28	-2.60	0.00933	0.02227
mmu-miR-7b-5p	29113.96	-0.70	0.27	-2.57	0.01024	0.02392
mmu-miR-1843b-5p	17879.89	-0.38	0.15	-2.57	0.01029	0.02392
mmu-miR-490-3p	1006.45	0.69	0.27	2.54	0.01093	0.02522
mmu-miR-493-5p	458.11	-0.51	0.20	-2.53	0.01130	0.02586
mmu-miR-700-3p	289.26	-0.60	0.24	-2.53	0.01140	0.02589
mmu-miR-204-5p	12837.22	-0.76	0.30	-2.52	0.01165	0.02608
mmu-miR-496a-3p	522.95	-0.70	0.28	-2.52	0.01166	0.02608
mmu-miR-24-1-5p	584.06	-0.43	0.17	-2.50	0.01246	0.02754
mmu-miR-26b-5p	9299.13	-0.40	0.16	-2.50	0.01250	0.02754
mmu-miR-194-5p	1781.41	-0.33	0.13	-2.48	0.01314	0.02873
mmu-miR-99a-5p	234379.56	-0.50	0.20	-2.45	0.01445	0.03136
mmu-miR-450a-5p	330.81	0.47	0.19	2.43	0.01504	0.03239
mmu-miR-148b-5p	272.71	0.52	0.21	2.41	0.01617	0.03458
mmu-miR-329-5p	4059.20	-0.29	0.12	-2.40	0.01662	0.03528
mmu-miR-344c-3p	384.00	-0.63	0.26	-2.37	0.01773	0.03738
mmu-miR-139-3p	1714.50	-0.42	0.18	-2.32	0.02038	0.04266
mmu-miR-378c	1923.60	0.38	0.17	2.26	0.02387	0.04960

Supplementary Table 4: List of Primers

Name of Primer	Forward	Reverse

AChE-S	CTGAACCTGAAGCCCTTAGAG	CCGCCTCGTCCAGAGTAT
AChE-R	GAGCAGGGAATGCACAAG	GGGGAGGTGGAGAAGAGAG
P300	TTCAGCCAAGCGGCCTAAA	CGCCACCATTGGTTAGTCCC
SIRT1	AGTTCCAGCCGTCTCTGTGT	CTCCACGAACAGCTTCACAA
HB-EGF	GGCCTCCTGTAATTGCTCTG	GCTCACTCGATCCTGCTTTC
MCP-1	AAAACACGGGACGAGAAACCC	ACGGGAACCTTTATTAACCCCT
IL18	GTGAACCCCAGACCAGACTG	CCTGGAACACGTTTCTGAAAGA
1L-6	TGAGAAAAGAGTTGTGCAATGG	GGTACTCCAGAAGACCAGAGG
TNFα	TAGCCCACGTCGTAGCAAAC	GCAGCCTTGTCCCTTGAAGA
IL1β	TGCCACCTTTTGACAGTGATG	TGATGTGCTGCTGCGAGATT
